# Identification and Toxigenic Potential of Fungi Isolated from *Capsicum* Peppers

**DOI:** 10.3390/microorganisms7090303

**Published:** 2019-08-30

**Authors:** Gabriel Kojo Frimpong, Adedotun Adeyinka Adekunle, Oluwatoyin Temitayo Ogundipe, Manoj Kumar Solanki, Sudharsan Sadhasivam, Edward Sionov

**Affiliations:** 1 Department of Botany, University of Lagos, Akoka 100213, Lagos, Nigeria; 2 Biotechnology and Nuclear Agriculture Research Institute, Ghana Atomic Energy Commission, Legon-Accra, Ghana; 3 Department of Food Quality and Safety, Institute for Postharvest and Food Sciences, The Volcani Center, Agricultural Research Organization, Rishon LeZion 7528809, Israel

**Keywords:** pepper, spoilage fungi, mycotoxins, HPLC

## Abstract

*Capsicum* peppers are among the most popular horticultural crops produced and consumed worldwide. This study aimed to assess the occurrence of spoilage fungi responsible for post-harvest losses in the most common varieties of *Capsicum* peppers collected from retail markets in Nigeria and Ghana. Forty fungal isolates belonging to 7 families, 8 genera, and 17 species were identified on the basis of morphology, culture characteristics, and DNA sequencing of the internal transcribed spacer (ITS) region. *Aspergillus* spp. (42.5%), *Fusarium* spp. (22.5%), and *Colletotrichum* spp. (15%) were found to be the predominant fungal pathogens. Furthermore, potential ability of the isolated mycotoxigenic fungi to produce some major mycotoxins was analyzed using high-performance liquid chromatography (HPLC). Among the 22 isolates analyzed, 11 strains belonging to the genera of *Aspergillus*, *Fusarium,* and *Penicillium* were found to be able to produce mycotoxins, such as aflatoxin B1, gliotoxin, deoxynivalenol, and citrinin. A better understanding of the role of fungal contaminants in pepper fruits, especially the prevalence of mycotoxigenic fungi and their associated mycotoxigenic potential, will assist in the development of management strategies to control mycotoxin contamination and to reduce toxicological risks related to pepper consumption by humans and animals.

## 1. Introduction

The *Capsicum* genus, which includes more than 30 species of flowering pepper plants, belongs to one of the most important families known as *Solanaceae*. Although developed countries continue to be the main producers of pepper crops, its cultivation provides an important source of income for small producers in many developing countries. Pepper is one of the most important vegetable crops contributing to significant foreign exchange earnings in Sub-Saharan Africa [1]. However, the incidence of pre- and post-harvest diseases has become a major constraint for the growers. Such as any other agricultural crops, *Capsicum* peppers are also susceptible to fungal infection and subsequent contamination with mycotoxins, which are toxic chemical products, formed as secondary metabolites by filamentous fungi. Several studies have described high frequencies of mycotoxigenic fungal strains in *Capsicum* fruits [2,3,4,5,6,7,8,9]. Historically, toxigenic fungi in crops have been divided into two distinct classes: “Field fungi”, which invade and produce their toxins before harvest, and “storage fungi”, which become a problem after harvest [10]. Many species of *Alternaria*, *Fusarium*, *Cladosporium,* and *Rhizopus* are associated with fungal infections of the growing plants in the field, while *Aspergillus* and *Penicillium* species are the predominant pathogens found in a high frequency of isolation from *Capsicum* by-products during the harvest and post-harvest stages, including drying, product transportation, and marketing (e.g., factory production, restaurants, retail markets) [3,11,12,13,14]. Furthermore, it has been reported that climatic conditions in tropical or sub-tropical regions, such as high temperature, humidity, and rainfall, contribute to the high fungal burden and mycotoxin contamination in pepper samples [15].

Most mycotoxins are chemically and thermally stable and cannot be destroyed during most food processing operations. Mycotoxins have become an important issue in relation to the food safety requirements for international marketing of agri-food commodities for human and animal consumption [16]. According to a previous study on co-occurrence of mycotoxins in *Capsicum* [13], pepper products can be contaminated with aflatoxins, ochratoxin A, fumonisins, zearalenone, trichothecenes, and patulin. Among those, aflatoxins and ochratoxin A are considered the most abundant and commonly found toxins in *Capsicum* peppers [16]. These compounds can cause a variety of adverse health effects, including carcinogenic and immunosuppressive activities [17,18]. 

In order to minimize microbial contamination and to improve the food safety standards, the Food and Agriculture Organization (FAO) and the World Health Organization (WHO) encourage government agencies to conduct microbiological risk assessment of foods and to provide data collection and documentation for improving regulations [19]. To the best of our knowledge, this is the first report supplying data on fungal contaminants in pepper fruits collected from several retail markets in Ghana and Nigeria. The objective of the present study was to provide accurate data on molecular identification and mycotoxigenic potential of fungal pathogens isolated from the peppers, which would assist in the development of management strategies to overcome mycotoxin contamination, minimize mycotoxicological risks of *Capsicum* peppers, and reduce the hazard to animal and human health. 

## 2. Materials and Methods

### 2.1. Sample Collection and Isolation of Fungi 

A total of 130 pepper fruits were collected from several retail markets and shopping centers located in Lagos, Nigeria and Accra, Ghana, between October 2016 to April 2018. Shombo, Rhodo, and Nsukka yellow were pepper varieties sampled from Lagos, while Yellow sisi, Makopa, Touto, and Kpakpo shito pepper varieties were sampled from Accra. The collected fruits were kept in sterile plastic bags during transport to the laboratory and, on the same day, were washed under running water and surface sterilized as follows: With 70% ethanol for 1 min, rinsed with sterile water, followed by immersion in 1% sodium hypochlorite for 1 min and two rinses with sterile distilled water. Segments (3–5 mm) of tissues from the margins of the infected areas were cut out with a sterile scalpel and placed on potato dextrose agar (PDA) (BD Difco, Sparks, MD, USA) supplemented with chloramphenicol (25 µg/mL) to discourage bacterial contamination. The plates were incubated at 28 °C for 3–5 days. Fungal isolates were transferred singly to PDA plates and subcultured twice to obtain pure cultures. 

### 2.2. Morphological and Molecular Identification of the Fungi

Morphological identification of the isolated fungi was carried out according to the Atlas of Clinical Fungi [20]. The following characters of the cultures were assessed by eye and microscopic examination: Mycelial growth and morphology, colony morphology (PDA), macroconidia, microconidia, and chlamydospores. Because of incomplete morphological identification, molecular identification of all fungal isolates was performed. Fungal genomic DNA was isolated from lyophilized mycelial mats grown overnight in potato dextrose broth (PDB) medium. Lyophilized mycelial mats were pulverized with 5 mL of 3-mm-diameter glass beads in a disposable 50-mL conical centrifuge tube. The DNA was extracted using a lysis buffer containing hexadecyltrimethylammonium bromide (CTAB) as described by Sadhasivam et al. [21]. The purity and quantity of the extracted DNA were determined using a NanoDrop One spectrophotometer (Thermo Scientific, Wilmington, DE, USA). The internal transcribed spacer (ITS) of the ribosomal RNA (rRNA) gene region in fungi was amplified using primers ITS1 (5′-TCCGTAGGAACCTGCGG-3′) and ITS4 (5′-TCCTCCGCTTATTGATATGC-3′). The PCR mixture (40 µL) contained 20 µL 2× DreamTaq green PCR mastermix (Thermo Fisher Scientific, Vilnius, Lithuania), 1 mL of each primer (5 mM), and 1 mL of DNA template. Nuclease-free water replaced template DNA in negative controls. PCR products were purified and sequenced through standard Sanger sequencing; sequences were identified via BLAST matches (Basic Local Alignment Search Tool, https://blast.ncbi.nlm.nih.gov/Blast.cgi) on the National Center for Biotechnology Information (NCBI) database, and a phylogenetic tree was constructed with the neighbor-joining method of Saitou and Nei [22]. The evolutionary analyses were conducted with 2000 bootstrap replications using the MEGA X software package [23]. Based on sequencing data, diversity index and principal component analyses were accomplished.

### 2.3. Pathogenicity Test

The pathogenicity test was performed by wound/drop methods described by Oo et al. [6]. The conidial suspension of the isolated fungi was prepared by adding 10 mL of sterilized distilled water onto the surface of the PDA plate, and spores were harvested with gentle brushing. Spore concentration was adjusted to 1 × 10^6^ conidia/mL using a hemocytometer prior to inoculation. Healthy pepper fruits of all varieties were washed in sterile distilled water and surface sterilized as described above. Then, the fruits were dried and placed on sterilized paper in moistened clean boxes. Sterile toothpicks were used to make wound in each fruit and 10 µL of conidial spore (1 × 10^6^ conidia/mL) inoculated into the wounding place. All control fruits were treated with 10 μL of sterilized distilled water. All boxes with inoculated fruits were incubated at 28 °C for 7 days, and disease symptoms were recorded. To conform the Koch’s postulates, the diseased fruits symptoms were compared with original symptoms, and the fungi were re-isolated from inoculated fruits following the above mention procedure. The inoculation experiments were replicated three times, and each replicate included three pepper fruits.

### 2.4. Mycotoxins Analysis

Aflatoxin (B1) was extracted based on the method described by Gell and Carbone [24], with some modifications. For the AFB1 extraction, 1.5 g of agar plug with fungal mycelia was crushed in 1.5 mL of chloroform and vortexed for 10–15 min. The upper phase was discarded and the lower chloroform phase was dried at 50 °C under a stream of gaseous nitrogen. The dried samples were reconstituted in 1 mL of methanol. Then, 200 µL of the reconstituted sample was derivatized with 300 µL of trifluoroacetic acid solution (70% water, 20% trifluoroacetic acid, and 10% acetic acid) for 20 min at 65 °C. After incubation, 500 µL of water was added to the reacted samples and vortexed vigorously for 10 s. Then the samples were filtered using 0.22 µm PTFE membrane filter, and quantitatively analyzed by injection of 20 µL into reverse phase UHPLC system (Waters ACQUITY Arc^TM^, FTN-R, Milford, MA, USA) with a gradient elution of 80% water and acetonitrile (20%) at 0.5 mL/min through a Kinetex 2.6 µm XB-C_18_ (100 × 2.1 mm) with a security guard column C18 (4 × 2 mm) (Phenomenex, Torrance, CA, USA). The column temperature was maintained at 35 °C. AFB1 peak was detected with a fluorescence detector (excitation at 365 nm and emission at 455 nm) and quantified by comparing with calibration curves of the standard mycotoxin for all experiments (Fermentek, Jerusalem, Israel).

Deoxynivalenol and ochratoxin A were extracted followed by a modified protocol [25,26]. A 1.5 g of agar plug with fungal mycelia was extracted with 1.5 mL of methanol and shaken for 30 min in an orbital shaker (300 rpm). The samples were centrifuged at 8000 rpm for 10 min. The extraction solutions were filtered through a syringe filter (PTFE 0.22 μm). Deoxynivalenol and ochratoxin A were analyzed by the above-mentioned instrument and column with a mobile phase consisting of 0.1% acetic acid and acetonitrile (80:20) for deoxynivalenol, and acetonitrile:water:acetic acid (99:99:2, *v/v/v*) for ochratoxin A at 0.5 mL/min. The column temperature was maintained at 30 °C. Deoxynivalenol peak was detected with photodiode array detector (219 nm) and the ochratoxin A peak was detected with a fluorescence detector (excitation at 330 nm and emission at 450 nm). 

Gliotoxin and citrinin were extracted with a modified protocol based on the method described by Guo et al. [27] and Pena et al. [28]. An agar plug of 1.5 g with fungal mycelia was transferred to a clean glass tube containing 1.5 mL of chloroform and crushed. After vortexing the extraction solution for 10–15 min, the samples were kept under shaking for 30 min in an orbital shaker (300 rpm) and centrifuged for 10 min at 8000 rpm. The supernatants were discarded and the chloroform layer was evaporated to dryness under a stream of gaseous nitrogen at 50 °C. The residues were redissolved in 1 mL of methanol and filtered through a syringe filter (PTFE 0.22 μm). The mobile phase of gliotoxin consisted of 75% (1% acetic acid in water) and 25% of acetonitrile, and the citrinin mobile phase consisted of acetified water (adjusted pH 2.5 with acetic acid) and acetonitrile (50:50). The toxins were analyzed using the above instrument and column with 0.5 mL/min flow rate. The column temperature was maintained at 30 °C. Gliotoxin was detected with a photodiode array detector (254 nm) and citrinin was detected with a fluorescence detector (331 nm excitation, 500 nm emission). 

## 3. Results and Discussion

**Figure 1:**
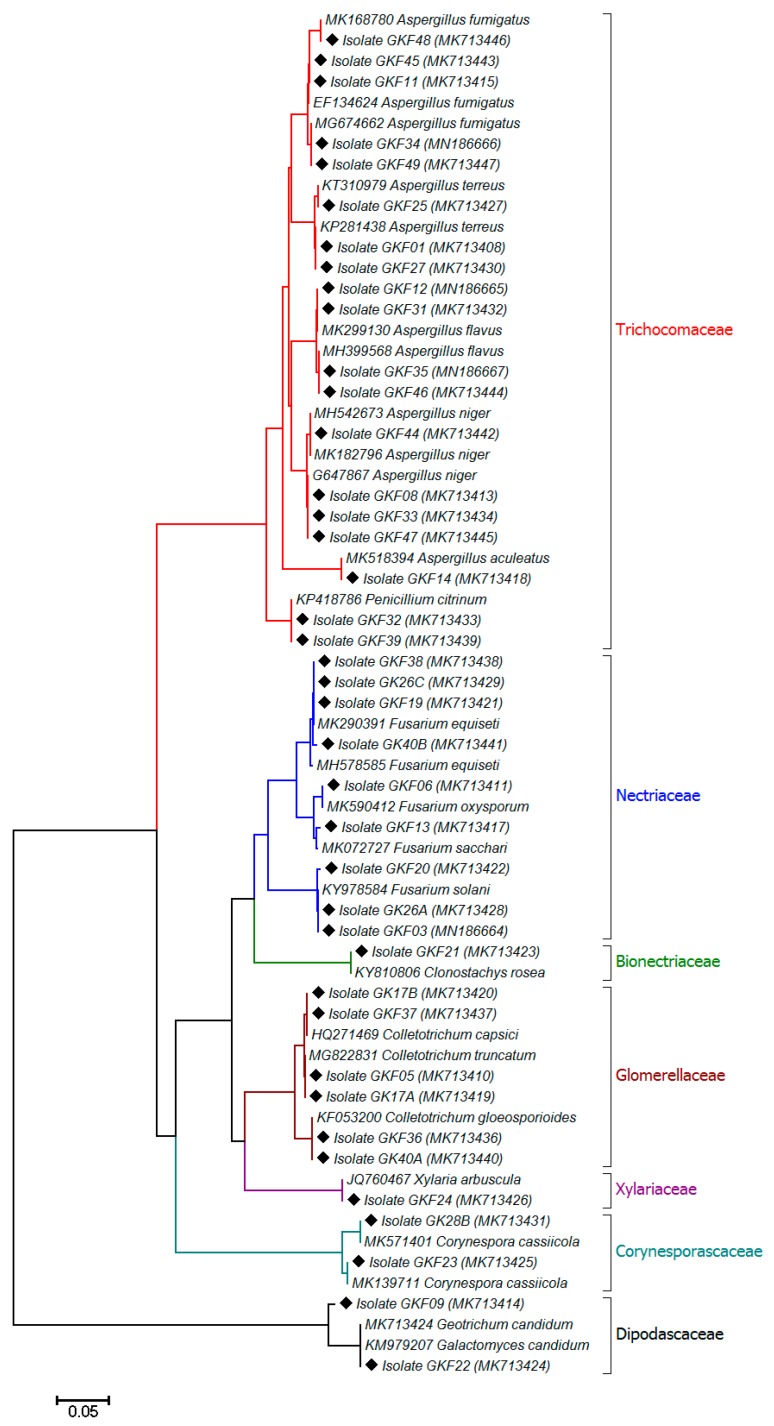
Phylogenetic analysis of ITS gene sequences of fungal pathogens isolated from *Capsicum* pepper. The fungal strains analyzed in the current study are shown by a diamond with their isolate code and accession number in parentheses. Sequences derived from the database are shown with their accession number and organism name. The tree was constructed using a Kimura 2-parameter distance with the neighbor-joining method. Bootstrap values (1000 time repeats) are used and the scale bar indicates 5 changes per 100 nucleotide positions. All positions containing gaps and missing data were eliminated.

A relatively high fungal diversity was observed across the analyzed pepper varieties. A total of 8 genera and 17 different fungal species were isolated from the pepper fruits. Phylogenetic analysis of ITS rRNA gene region sequences indicated that all sequences of the isolated fungi were assigned to the phylum *Ascomycota*, which were mainly represented by the fungal family of *Trichocomaceae* (47.5%), followed by the families of *Nectriaceae* (22.5%), *Glomerellaceae* (15%), *Corynesporascaceae* (5%), *Dipodascaceae* (5%), *Bionectriaceae* (2.5%), and *Xylariaceae* (2.5%) (Figure 1). Forty fungal isolates, which were cultured from the pepper fruits, mainly consisted of the genera *Aspergillus*, followed by *Fusarium* and *Colletotrichum*; several species of *Colletotrichum* and other genera such as *Corynespora*, *Xylaria*, *Clonostachys,* and *Geotrichum* were also found in pepper samples and identified based on morphological features (colony color, texture, microscopic observation) and sequencing analysis (Tables S1 and S2). Overall, the mycotoxigenic species, such as *Aspergillus flavus*, *Aspergillus niger*, *Aspergillus fumigatus*, *Penicillium citrinum*, *Fusarium solani,* and *Fusarium equiseti* were the most abundant fungi isolated from the peppers (Table S3). Findings in the current study are consistent with previous studies that have found high levels of fungal contamination in peppers, mainly by mycotoxigenic species belonging to *Aspergillus*, *Fusarium*, and *Penicillium* [16]. Moreover, it has been previously reported that several *Aspergillus* and *Fusarium* species are responsible for the post-harvest deterioration of fresh *Capsicum* peppers collected from different markets and farm lands in Nigeria, which were surveyed together with some edible fruits and vegetables [29,30]. 

**Figure 2:**
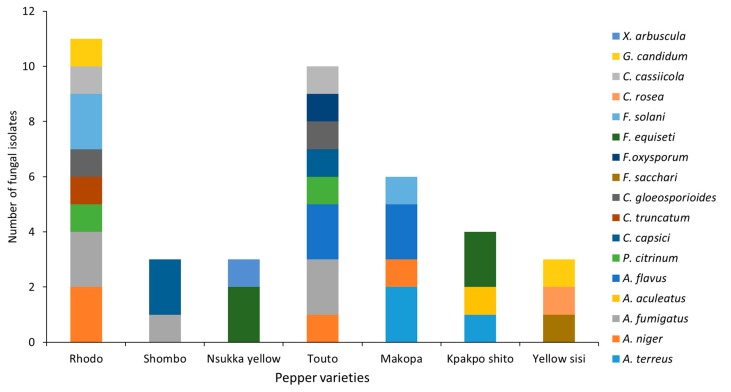
Fungal distribution among the most common pepper varieties collected from retail markets.

Figure 2 clearly demonstrates that among the pepper fruits surveyed in the current study, varieties Rhodo, Touto, and Makopa were found to be most sensitive to fungal infection, and *A. niger*, *A. flavus*, *A. fumigatus*, *P. citrinum*, *Fusarium oxysporum*, *F. equiseti*, *Colletotrichum gloeosporioides,* and *Corynespora cassiicola* were the most common fungi isolated from the peppers. *C. cassiicola* is known as a pathogen of many agricultural crop plants and causes dark-brown spots on leaves, fruits, and stems in several hosts, such as cotton, cucumber, tomato, papaya, soybean, eggplant, okra, and other important economic crops [31,32,33,34,35]. *C. cassiicola* has been reported previously to infect sweet and hot pepper plants in Japan and China, respectively [36,37]. To the best of our knowledge, this is the first report on the presence of *C. cassiicola* on *Capsicum* fresh pepper fruits that were collected for the current study from retail markets in Nigeria and Ghana. Since different varieties have different sensitivity to fungal infection, it is noteworthy that fungal contamination was considerably lower in pepper varieties such as Shombo, Nsukka yellow, Kpakpo shito, and Yellow sisi, where *Aspergillus aculeatus*, *Xylaria arbuscula*, *Geotrichum candidum*, *Clonostachys rosea,* and *Fusarium sacchari* were the predominant isolated species (Figure 2). A low fungal burden in these pepper fruit samples could also be related to the presence of antifungal compounds, which would be restricting the growth and proliferation of these microorganisms. A few studies have been conducted to determine the antifungal properties of *Capsicum* species, which is mainly attributed to the pungent compound capsaicin, present in large amounts in different varieties of *Capsicum* [38,39]. Seven days after inoculation, disease symptoms caused by fungi appeared in all of the inoculated pepper fruits, and these symptoms were the same as those observed on the peppers collected from retail markets. The incidence of the disease caused by fungal pathogens in inoculated *Capsicum* fruits was 100% in each of the three replicates. No symptoms were observed on the control fruits. The morphological characteristics of the re-isolates were identical with the original isolates. The results showed that the isolated fungi were the main causative agents of fungal infections in pepper fruits. 

In the current study, potentially mycotoxigenic species, such as *A. flavus*, *A. niger*, *A. fumigatus*, *P. citrinum*, *F. solani* and *F. equiseti* were distributed on most of the tested pepper varieties, with higher occurrence in Rhodo, Touto and Makopa varieties. Among four *A. flavus* species isolated from the pepper fruits only one was able to produce aflatoxin B1 (AFB1) in culture media (Table 1). Although *A. flavus* is one of the most common producers of aflatoxins not all isolates of this species are aflatoxigenic. It has been estimated that only about 30–40% of *A. flavus* isolates are able to produce aflatoxins [40]. Aflatoxins, which are secondary metabolites produced by various *Aspergillus* species belonging to *Aspergillus* section *Flavi*, found in a range of food/feed products, especially those originating from tropical/sub-tropical regions [16]. The study conducted by Singh and Cotty [15] evaluated the occurrence of AFB1 in red chilli peppers collected from markets across the USA and Nigeria. These authors found that AFB1 concentrations were significantly higher in Nigerian pepper compared to those purchased in the USA. The International Agency for Research on Cancer (IARC) defined aflatoxins as carcinogenic (Group 1) and potentially carcinogenic to human (Group 2B) [18].

All five *A. fumigatus* strains isolated from the tested peppers were able to produce gliotoxin in vitro (Table 1), which is a highly toxic metabolite that has potent immunosuppressive, genotoxic, cytotoxic, and apoptotic effects [41]. Very few studies have reported regarding *A. fumigatus* contamination in peppers [30,42], and none have considered the adverse effects of gliotoxin, which could influence consumer health.

Of the potentially ochratoxigenic *Aspergillus* species, *A. niger* was the only species isolated from different varieties of the tested peppers (Figure 2). None of the four *A. niger* strains isolated from *Capsicum* were able to produce OTA in culture media (Table 1). OTA is the second most common mycotoxin (after aflatoxins) found in pepper samples [16]. A global health issue arose from OTA contamination of a wide range of food commodities, due to its immunosuppressive, nephrotoxic, and carcinogenic potential. This mycotoxin is categorized by IARC as possibly carcinogenic to humans under Group 2B carcinogen [18]. Although it was not determined in this study, the contamination by OTA has previously been reported in commercialized *Capsicum* pepper samples [43,44].

A relatively high level of deoxynivalenol (DON) produced by *Fusarium* species was also observed in culture media (Table 1). Out of nine cultured *Fusarium* isolates, three *F. solani* strains and one strain of *F. equiseti* were found to produce DON at average concentration levels ranging from 3780.2–8127.8 ng/g agar. These findings are consistent with other studies, where mycotoxigenic *F. solani* and *F. equiseti* are found among the fungal species isolated from fresh *Capsicum* pepper and by-products [13,30]. *F. oxysporum* and *F. sacchari*, which were among the fungi isolated from the tested peppers, are considered to only produce fusaric acid [45], therefore they were not examined for DON production in the current study. 

*Penicillium* species may constitute a serious problem of spoilage and mycotoxin contamination of *Capsicum* products. In the current study, potential citrinin producer *P. citrinum* isolates were cultured from the pepper samples. Only one of the tested isolates was able to produce citrinin in vitro (Table 1). The occurrence of citrinin, DON, and gliotoxin in pepper is an important issue that need to be studied in more detail.

In summary, our study of *Capsicum* pepper-associated mycobiota demonstrated that *Aspergillus* spp., *Fusarium* spp., and *Penicillium* spp. were predominant in the samples. Some of these species had toxigenic potential and were able to produce significant amounts of mycotoxins, such as AFB1, gliotoxin, DON, and citrinin, under the test conditions. The high rates of fungal contamination and high mycotoxin-producing capacity of the mycotoxigenic species observed in this study require an effective food safety management of pepper crop production, processing, and storage before release of this food commodity to the local market. 

## Figures and Tables

**Table 1:** Mycotoxigenic capacity of fungi isolated from fresh peppers.

Isolate	Species	Mycotoxins (ng/g agar ± SD) ^a^				
		AFB1	OTA	Gliotoxin	DON	Citrinin
GKF12	*A. flavus*	49.3 ± 3.2	-	-	-	-
GKF31	*A. flavus*	nd	-	-	-	-
GKF35	*A. flavus*	nd	-	-	-	-
GKF46	*A. flavus*	nd	-	-	-	-
GKF08	*A. niger*	-	nd	-	-	-
GKF33	*A. niger*	-	nd	-	-	-
GKF44	*A. niger*	-	nd	-	-	-
GKF47	*A. niger*	-	nd	-	-	-
GKF11	*A. fumigatus*	-	-	1015.55 ± 30.3	-	-
GKF34	*A. fumigatus*	-	-	1917.17 ± 61.5	-	-
GKF45	*A. fumigatus*	-	-	1260.13 ± 42.6	-	-
GKF48	*A. fumigatus*	-	-	1394.98 ± 38.5	-	-
GKF49	*A. fumigatus*	-	-	1318.23 ± 41.1	-	-
GKF03	*F. solani*	-	-	-	8127.85 ± 196.2	-
GKF20	*F. solani*	-	-	-	6744.66 ± 150.7	-
GK26A	*F. solani*	-	-	-	3903.92 ± 109.3	-
GKF19	*F. equiseti*	-	-	-	3780.28 ± 93.01	-
GK26C	*F. equiseti*	-	-	-	nd	-
GKF38	*F. equiseti*	-	-	-	nd	-
GK40B	*F. equiseti*	-	-	-	nd	-
GKF32	*P. citrinum*	-	-	-	-	273.25 ± 13.5
GKF39	*P. citrinum*	-	-	-	-	nd

^a^ Average value of three replicates; nd, not detected (below detection limit).

## Supplementary Materials

The following are available online at https://www.mdpi.com/2076-2607/7/9/303/s1. Table S1: Morphological characterization of fungal pathogens isolated from the fresh pepper fruits; Table S2: Sequencing results of fungal pathogens of pepper fruits; Table S3: Fungal pathogens isolated from different varieties of *Capsicim* pepper collected in Nigeria and Ghana.
