# Supplementary material for: Identification and Toxigenic Potential of Fungi Isolated from Capsicum Peppers
Source: Microorganisms. 2019 Aug 30;7(9):303. doi: 10.3390/microorganisms7090303 (PMC6780498; doi:10.3390/microorganisms7090303)
Supplement: Supplementary file 1 [file microorganisms-07-00303-s001.pdf]

microorganisms

MDPI

Supplementary Material

Table 1. Phenotypic characterization of fungal pathogens isolated from the fresh pepper fruits.

Isolates	Colony characteristics, microscopy, and morphology	Organism
GKF01, GKF08, GKF11, GKF12, GKF14, GKF25, GKF27, GKF31, GKF33, GKF34, GKF35, GKF44, GKF45, GKF46, GKF47, GKF48, GKF49	Colonies' color of these isolates ranges from green to dark-brown or black. Conidiophores erect, unbranched, with swollen apical vesicles. Conidial heads columnar, uniseriate; vesicles spherical.	*Aspergillus*
GKF32, GKF39	The mycelia color of these isolates is green to greyish-orange. Colonies velutinous to floccose. Conidiophores stipes smooth-walled, biverticilate. Conidia globose or ellipsoidal, smooth-walled, produced in long columns.	*Penicillium*
GKF03, GKF13, GKF06, GKF19, GK26C, GKF38, GK40B, GKF20, GK26A	These isolates' mycelia have characteristically white or pink color. The presence of micro and macroconidia was observed under light microscope. Macroconidia are spindle-shaped, straight to slightly curved or sickle-shaped. Chlamydospores are present in several species.	*Fusarium*
GKF05, GK17A, GK17B, GKF37, GKF36, GK40A	The colonies have large, dark-walled stromatic structures, and are present in the cultures forming perithecia, often embedded in agar, with their ascospores strongly curved and typically tapering towards the ends. Conidiophores have hyaline, fusiform, spindle shapes, with acute apices.	*Collectotrichum*
GKF23, GK28B	The colonies are whitish-grey in color and darken to olive green. Conidia are clavate or cylindrical, straight or curved, smooth, septate, pale olivaceous brown or catenate, and sometimes pseudosepta, especially in immature conidia.	*Corynespora*
GKF09, GKF22	These isolates' colonies are flat, white to cream, dry, and finely suede-like with no reverse pigment. Hyphae are hyaline, septate, branched, and break up into chains of hyaline, smooth, one-celled, sub-globose to cylindrical arthroconidia.	*Geotrichum*
GKF21	These isolates have cottony colonies with a powdery surface which turns to light orange color at maturity. Hyaline conidia with a finely rough surface, broadly rounded, with lateral hilum.	*Clonostachys*

GKF24	The mycelia color of the isolates is a dark-grey to brown, becoming dark-black at maturity. The colonies are sub-cylindric at first, becoming flattened. Upper branches appear powdered white, finally tipped black when mature, stalk black and hairy.	*Xylaria*

Table 2. Sequencing results of fungal pathogens of pepper fruits.

Isolates	Relative organism	Coverage (%)	Similarity (%)	Accession no.
GKF01	*Aspergillus terreus*	100	100	MK713408
GKF03	*Fusarium solani*	100	100	MN186664
GKF05	*Colletotrichum capsici*	100	100	MK713410
GKF06	*Fusarium oxysporum*	100	100	MK713411
GKF08	*Aspergillus niger*	100	100	MK713413
GKF09	*Geotrichum candidum*	100	100	MK713414
GKF11	*Aspergillus fumigatus*	100	100	MK713415
GKF12	*Aspergillus flavus*	100	100	MN186665
GKF13	*Fusarium proliferatum*	100	100	MK713417
GKF14	*Aspergillus aculeatus*	100	100	MK713418
GF17A	*Colletotrichum truncatum*	100	100	MK713419
GF17B	*Colletotrichum capsici*	100	100	MK713420
GKF19	*Fusarium equiseti*	100	100	MK713421
GKF20	*Fusarium solani*	100	100	MK713422
GKF21	*Clonostachys rosea*	100	100	MK713423
GKF22	*Galactomyces candidum*	100	100	MK713424
GKF23	*Corynespora cassiicola*	100	100	MK713425
GKF24	*Xylaria arbuscula*	99	97	MK713426
GKF25	*Aspergillus terreus*	100	100	MK713427
GK26A	*Fusarium solani*	100	100	MK713428
GK26C	*Fusarium equiseti*	99	100	MK713429
GKF27	*Aspergillus terreus*	99	100	MK713430
GF28B	*Corynespora cassiicola*	99	100	MK713431
GKF31	*Aspergillus flavus*	100	100	MK713432
GKF32	*Penicillium citrinum*	99	100	MK713433
GKF33	*Aspergillus niger*	99	100	MK713434
GKF34	*Aspergillus fumigatus*	100	100	MN186666
GKF35	*Aspergillus flavus*	100	100	MN186667

GKF36	*Colletotrichum gloeosporioides*	99	100	MK713436
GKF37	*Colletotrichum truncatum*	100	100	MK713437
GKF38	*Fusarium equiseti*	100	100	MK713438
GKF39	*Penicillium citrinum*	99	100	MK713439
GK40A	*Colletotrichum gloeosporioides*	99	100	MK713440
GK40B	*Fusarium equiseti*	100	100	MK713441
GKF44	*Aspergillus niger*	100	100	MK713442
GKF45	*Aspergillus fumigatus*	100	100	MK713443
GKF46	*Aspergillus flavus*	99	100	MK713444
GKF47	*Aspergillus niger*	98	100	MK713445
GKF48	*Aspergillus fumigatus*	99	100	MK713446
GKF49	*Aspergillus fumigatus*	100	100	MK713447

Sequences were identified via BLAST matches (Basic Local Alignment Search Tool, https://blast.ncbi.nlm.nih.gov/Blast.cgi) on the National Center for Biotechnology Information (NCBI) database.

Table 3. Fungal pathogens isolated from different varieties of *Capsicim* pepper collected in Nigeria and Ghana.

Genus	Species	Distribution of fungal species across the pepper samples						
		Rhodo	Shombo	Nsukka yellow	Touto	Makopa	Kpakpo shito	Yellow sisi
Aspergillus	*A. terreus*	0	0	0	0	2	1	0
	A. niger	2	0	0	1	1	0	0
	A. fumigatus	2	1	0	2	0	0	0
	A. aculeatus	0	0	0	0	0	1	0
	A. flavus	0	0	0	2	2	0	0
Penicillium	*P. citrinum*	1	0	0	1	0	0	0
Colletotrichum	*C. capsici*	0	2	0	1	0	0	0
	C. truncatum	1	0	0	0	0	0	0
	C. gloeosporioides	1	0	0	1	0	0	0
Fusarium	*F. sacchari*	0	0	0	0	0	0	1
	F.oxysporum	0	0	0	1	0	0	0
	F. equiseti	0	0	2	0	0	2	0
	F. solani	2	0	0	0	1	0	0
Clonostachys	*C. rosea*	0	0	0	0	0	0	1
Corynespora	*C. cassiicola*	1	0	0	1	0	0	0
Geotrichum	*G. candidum*	1	0	0	0	0	0	1
Xylaria	*X. arbuscula*	0	0	1	0	0	0	0
